# Fibrotic remodeling in the NOD/ShiLtJ mouse model of Sjögren’s disease: insights from single-cell transcriptomics and AI-driven ECM quantification

**DOI:** 10.3389/fimmu.2026.1779014

**Published:** 2026-07-20

**Authors:** Jennifer M. Morrissey, Deirdre A. Nelson, Li Chen, Mathieu Petitjean, Joey R. Tavarez, Amber L. Altrieth-Flagg, Nicholas L. Moskwa, Renae Williams-Atkinson, Ben Fowler, Rafael Pena, Kennedi Weston, Nikhita Kumar, Nathan Aist, Melinda Larsen

**Affiliations:** 1 Molecular, Cellular, Developmental, and Neural Biology Graduate Program, State University of New York, University at Albany, Albany, NY, United States; 2 Department of Biological Sciences and The RNA Institute, State University of New York, University at Albany, Albany, NY, United States; 3 PharmaNest Inc., Princeton NJ, United States; 4 Imaging Facility and Histology Core, Oklahoma Medical Research Foundation, Oklahoma City, OK, United States; 5 Norton College of Medicine, State University of New York, Upstate Medical University, Syracuse, NY, United States

**Keywords:** extracellular matrix, fibrosis, nintedanib, NOD/ShiLtJ mouse, salivary gland, single-cell RNA sequencing, Sjögren’s disease

## Abstract

**Introduction:**

Sjögren's Disease (SjD) is an autoimmune disorder characterized by salivary gland hypofunction and lymphocytic infiltration, yet the contribution of fibrosis to glandular dysfunction remains unclear.

**Methods:**

We evaluated the NOD/ShiLtJ mouse as a model for salivary gland fibrosis and used it to examine the impact of antifibrotic therapy on extracellular matrix (ECM) remodeling. To assess therapeutic potential, we treated NOD/ShiLtJ female mice with nintedanib, an FDA-approved antifibrotic agent used to treat chronic fibrosing interstitial lung disease. Fibrosis was assessed using single-cell RNA sequencing combined with Picrosirius red staining and AI-assisted digital pathology quantification of collagen I patterning with FibroNest.

**Results:**

Single-cell RNA sequencing revealed that fibroblast populations in the submandibular and sublingual salivary glands of the female NOD/ShiLtJ mouse exhibited increased levels of ECM genes, including *Col1a1*, *Col1a2*, and *Col3a1*, relative to control mice. Consistent with these transcriptional changes, Picrosirius Red staining, combined with AI-assisted fibrosis quantification of collagen I patterns, demonstrated significant fibrotic remodeling in the periacinar ECM in submandibular glands of female NOD/ShiLtJ mice relative to controls. Notably, fibrosis severity strongly correlated with the diabetic phenotype characteristic of this strain, and both periductal and periacinar fibrosis progressed with age. An 8-week nintedanib treatment resulted in modest but measurable reductions across multiple fibrotic indices.

**Discussion:**

Collectively, these findings establish the NOD/ShiLtJ strain as a model of SjD-associated salivary gland fibrosis and provide proof-of-concept evidence supporting antifibrotic strategies as a potential therapeutic avenue for salivary gland dysfunction in SjD.

## 1 Introduction

SjD is a rheumatic autoimmune disease that occurs primarily in menopausal females that leads to chronic and severe dry eye and dry mouth. The dry mouth symptoms are ascribed to salivary hypofunction, which is defined by a decrease in saliva production (1), and leads to the subjective feeling of xerostomia (dry mouth), halitosis (bad breath), dysphagia (difficulty swallowing), dysphonia (hoarseness), dysgeusia (altered taste), burning sensations in the oral cavity, dental caries (cavities), oral candidiasis (fungal infections), gingivitis, and periodontitis (inflammation of the gums). These oral symptoms cause a significant decrease in quality of life for SjD patients (2). Although there are new strategies being investigated, there are currently few treatments available (2, 3).

While SjD is characterized by hyposalivation, the underlying cellular and molecular mechanisms for this pathology are not fully understood. However, it is known that the etiology of SjD is complex with risk factors including age, female sex, as well as genetic, epigenetic, environmental, and stochastic factors leading to dysregulation of both innate and adaptive immunity (4–6). Inflammation is generally inversely correlated with gland function, while fibrosis is less understood and less studied. Fibrosis is the excessive accumulation of extracellular matrix (ECM) within tissues. The ECM is a complex extracellular structural and regulatory network comprised of collagens, glycoproteins, glycosaminoglycans, growth factors, and other regulators including various proteases (7–9). These ECM molecules are critical in both development of the gland and for maintaining gland homeostasis in adults; however, their relationship with SjD development is incompletely understood.

Fibroblasts, which reside in the connective tissue of organs, are required contributors to development and homeostasis, in part through their production and remodeling of ECM (10). However, fibroblasts can also undergo conversion to a phenotype known as a myofibroblast, where they contribute to aberrant ECM production and remodeling to become drivers of fibrosis (11) and inflammation (12, 13). Tissue damage and wounding can promote a fibroblast to myofibroblast transition, and these myofibroblasts have enhanced ECM deposition and modification capacity to promote healing. However, when this repair process is not productive and the wound does not heal, fibrosis persists and expands causing tissue pathology and impaired organ function (14, 15). Fibroblasts from SjD patient’s salivary glands, show proinflammatory properties (16–18) and therefore are potential disease drivers of SjD.

Patients with suspected SjD are assessed using the 2016 ACR–EULAR Classification Criteria, which was jointly developed by the American College of Rheumatology (ACR) and the European League Against Rheumatism (EULAR). A classification of SjD requires a total score of 4 or greater from the weighted sum of scores from 4 objective criteria: anti-SSA antibodies, focus score, ocular tests, and salivary flow (19). Salivary fibrosis is relatively common in SjD patients and is observed in labial salivary glands from SjD patients from which focus scores are calculated (20). Although fibrosis is positively associated with focus scores independent of age and negatively correlated with gland function in SjD patients (20), it is not used as a diagnostic factor for SjD diagnosis. The correlations of fibrosis with salivary gland dysfunction and proinflammatory fibroblasts highlight fibrosis as an underappreciated potential disease metric for SjD.

The non-obese diabetic (NOD/ShiLtJ) mouse, which develops both diabetes and a Sjogren’s like disease, has been used as a model for SjD because it develops many features of the disease. Similar to humans, the mouse develops autoantibodies and progressive B cell-containing lymphocytic infiltrates in the salivary glands over time, primarily within the proximity of large ducts (21–24). Lymphocytic infiltration into the submandibular salivary gland (SMG) begins by 12 weeks, and progresses to involve many large lymphocytic foci by 18 to 22 weeks in the NOD/ShiLtJ SMG (21). Infiltrations are accompanied by a peri-ductal fibrotic response in NOD mice, as documented previously with trichrome staining (25, 26).

Anti-fibrotic drugs have been tested as therapeutics for many diseases with some degree of success. Nintedanib, one of three FDA-approved anti-fibrotic drugs, targets multiple tyrosine kinases, including platelet-derived growth factor receptors (PDGFRs), vascular endothelial growth factor receptors (VEGFR)-1 and 2, and fibroblast growth factor receptors (FGFR)-1–3 (27). Phase II and III pooled clinical trials, conducted using nintedanib (OFEV) in the treatment of idiopathic pulmonary fibrosis (IPF) showed significant improvement in treated patients when compared to placebo treated patients (28). In IPF, nintedanib reduced growth factor-stimulated migration and proliferation of fibroblasts and expression of markers for transforming growth factor beta (TGFβ)-induced transition of fibroblasts to myofibroblasts, which is a hallmark of fibrotic disease (29). Nintedanib has also shown efficacy in reducing fibrosis in mouse models for several conditions in different organs, including IPF (30), interstitial lung disease (ILD) (31), systemic sclerosis (skin) (32), and liver fibrosis (33). The primary target of nintedanib in these studies is assumed to be growth factor signaling in the myofibroblasts; however, in most studies direct targets were not confirmed. While fibrosis has been shown to impair gland function in several diseases including decreased lung function in IPF (34) and decreased kidney function in chronic kidney disease (CKD) (35), whether fibrosis is a causative factor in SjD related salivary gland hypofunction is unknown.

We hypothesized that fibroblast-driven fibrosis is partially responsible for SjD disease progression. To examine its relevance in a disease model for SjD, we examined the fibrotic response in NOD/ShiLtJ mouse submandibular glands and used AI-assisted quantification of Picrosirius Red (PSR)-stained SMGs to quantify changes in the patterning of collagen I detected by PSR staining. We evaluated treatment of NOD/ShiLtJ mice during SjD disease development with the fibrotic inhibitor nintedanib to provide insight into whether fibrotic inhibitors might promise as a therapeutic option for SjD patients.

## 2 Materials and methods

### 2.1 Animal husbandry

Female NOD/ShiLtJ mice (RRID: IMSR_JAX:001976) and outbred CD1 mice (Crl: CD1(ICR)) were maintained by the University at Albany per approved IACUC protocol. Mice were ear punched with unique identifiers after arrival at University at Albany, allowing blind data processing. Mice were housed 5 per cage and kept in a 12-hour light/dark cycle. Cages were cleaned every other day. Starting at 12 weeks, wet feed was provided daily. All procedures, tissue and fluid collections were performed per approved IACUC protocol. Mice were assessed daily/weekly for body condition and other general health characteristics, including dehydration. NOD/ShiLtJ mice that seemed dehydrated were given sterile subcutaneous saline injections as needed.

### 2.2 Cell isolation for RNA sequencing

Mouse SMGs from four mice each of NOD/ShiLtJ and CD-1 together with adjacent sublingual glands (SLGs) were harvested and pooled. The four glands in two dishes containing 1.6 mL of Hanks Buffered Saline Solution (HBSS) and 400 µL of 2X collagenase/hyaluronidase and were broken into lobules using forceps for 10 minutes. Immediately following, an equal volume of 1.6 U/mL dispase was added, and the tissue was incubated for 10 minutes at 37 °C. The suspension was triturated 100 times and again 100 times 5 minutes later. The suspension was transferred to a 15 mL conical tube and centrifuged at 450xg for 5 minutes. The supernatant was discarded, and the cells were resuspended in 5 mL of 1xHBSS/5mM EDTA and centrifuged at 10xg for 1 minute. The supernatant was transferred to a new 15 mL tube and centrifuged at 300xg for 8 minutes. The cells were resuspended in 1 mL of 0.5 mg/mL DNAse and left at room temperature for 2 minutes then resuspended in 5 mL of 1xHBSS/5 mM EDTA. The cells then underwent epithelial cell depletion and dead cell depletion as described previously (36).

### 2.3 scRNA-seq

Single cell RNA sequencing analysis was performed as described previously (36), except that sequencing was performed on the NextSeq500. The two datasets (NOD/ShiLtJ and CD1) were integrated prior to analysis. Marker genes to identify cell types and to quantify changes in fibroblast populations were the same as we described previously and are shown in the figures (36–38).

### 2.4 Nintedanib treatment

Nintedanib (Selleck, Houston, TX) or vehicle (0.5% hydroxyethyl cellulose) treatment was started at 16 weeks of age, where each group contained 8 female NOD/ShiLtJ mice. Mice were treated every other day via oral gavage until 24 weeks of age. The vehicle (0.5% hydroxyethyl cellulose solution) (Sigma-Aldrich, St. Louis, MO) was prepared in water and adjusted to a pH of 3 with 1M (1N) hydrochloric acid. 20 mg of nintedanib was dissolved in 3.3 mL of 0.5% hydroxyethyl cellulose solution by warming solution to approximately 37 °C and vortexing for 30 seconds and rolling on a shaker for at least 2 minutes. Individual aliquots were frozen until use at -20 °C. Nintedanib was prepared at a concentration of 60 mg/mL, and each mouse was dosed with 1.5 mg for each oral gavage.

### 2.5 Glucose measurement

Glucose measurements were performed at 20 and 24 weeks on NOD/ShiLtJ mice using the FreeStyle Glucometer and test strips (Abbott, Lake County, Illinois). Glucose readings were taken mid-morning from nonfasted mice. Tails were wiped clean with alcohol and dried with gauze. A 27G needle was used to prick the tail to cause a small bubble of blood on the surface, which was absorbed onto the strip. The Freestyle test strip was placed in the meter, and a reading was obtained and recorded.

### 2.6 Cryopreservation and cryosectioning

Mice were euthanized using carbon dioxide at a CO_2_ flow rate of 2.1 L/min. Both SMG and SLG glands were removed, keeping the SLG attached to the SMG and weighed on an analytical balance. Glands were collected at 16, 20, or 24 weeks of age, as indicated. Glands were fixed and cryopreserved as previously described (37). Slides were collected by systematic serial sectioning with 10 µm sections using a Leica Biosystems cryostat, Model CM1860 (Leica Biosystems, Deer Park, IL). Tissues were arrayed one per slide on 27 slides, until all 27 slides contained a section, and then the 28^th^ section was applied to slide 1, continuing until the entire tissue was sectioned. Sections were dried for 30 minutes at room temperature and stored at −80 °C.

### 2.7 Picrosirius red staining

Slides containing tissue sections from NOD/ShiLtJ or CD1 SMG/SLG were removed from the -80 °C freezer and placed in Bouin’s Fixative (Polysciences, Warrington, PA) at room temperature (RT) overnight. Slides were then placed in 60 °C pre-warmed Bouin’s fluid and incubated at 60 °C for 60 min. Slides were dipped in tap water 10 times, then placed in Picrosirius Red (PSR) stain (ScyTec Laboratories, Logan, Utah) for 15 min. PSR-stained slides were then washed in running tap water for 1 minute and then incubated in picric acid (New Commer Supply, Middleton, WI) for 40 minutes and isopropanol for 5 minutes. Slides underwent a series of dehydration steps and were dipped 5 times in each of the following solutions, 100% ethanol, and 100% ethanol. Slides were then dipped 10 times in a 1:1 mixture of 100% ethanol and xylene (Sigma-Aldrich, St. Louis, MO), and incubated in a xylene bath for 1 minute, followed by one final xylene bath for 1 minute. Coverslips were mounted with 20-40 µL of Permount mounting medium (Electron Microscopy Sciences, Hatfield, PA).

### 2.8 Slide imaging and digital pathology

Slides from tissue microarrays containing samples from NOD and CD1 mice (21) that were stained with PSR were imaged using an EVIDENT VS200 slide scanner (EVIDENT Scientific, Tokyo, Japan). The exported images were processed in Photoshop (Adobe, San Jose, CA). For the NOD and CD1 images that were quantified by Pharmanest, one slide from each animal containing 5–6 sections was digitized on a Whole Slide Imaging (WSI) Hamamatsu NanoZoomer, RS2.0 Scanner (Hamamatsu Photonics K.K., Bridgewater, NJ) at 40X (0.250 micron per pixel). Images were viewed using the Hamamatsu NDP.view2 Image viewing software U12388-01. Images were quantified with FIJI (39).

### 2.9 Digital pathology quantification of the fibrosis phenotype

A representative WSI digital image of a PSR-stained tissue that contained minimal rips or folds was selected from the central region of each gland and uploaded for Digital Pathology quantification with FibroNest™ (PharmaNest, Princeton, NJ), a cloud-based, high-resolution, single-fiber image analysis platform. FibroNest was used to quantify the fibrosis phenotype in the context of three complementary phenotypic layers for: (i) collagen deposition and structure features (12 traits), (ii) fiber morphometry (12 traits), and (iii) fibrosis architecture (7 traits to measure the organization of the fiber (40)).

Color normalization and standardization (41, 42) were performed to standardize the images. Each trait was quantified with 7 quantitative statistical parameters (qFTs) to account for severity, distortion, and variance, resulting in a total of 337 qFTs. Fibers were also classified into “fine” (simple skeleton or low node/branch ratio) and “assembled” (complex skeleton or high node/branch ratio). The dimensionality of the qFT dataset was automatically reduced by identifying the traits (principal qFTs) that exhibit a significant (p<0.05) and meaningful (>20%) relative difference (group average) between the CD1 group and the NOD/ShiLtJ group. The principal qFTs (140 in this dataset) were assembled into a normalized Phenotypic Fibrosis Composite Score, and their variation was visualized in the form of a heat map. Similarly, the principal qFT that are associated with a specific phenotype layer (Collagen Deposition, Morphometry of the Fibers and Architecture of the Fibrosis), or a specific class (Fine-FINE or Assembled fibers- ASSBLD), were assembled into specific sub-scores, for instance the Fibrosis Morphometric Composite Score for Assembled fibers. Differences between the vehicle- and nintedanib-treated SMGs for each qFT were calculated by Student’s t-test. A visual workflow of the method and the full list and nomenclature describing all of the 336 fibrosis qFTs as previously described (43).

## 3 Results

### 3.1 NOD/ShiLtJ fibroblasts and macrophages show a pro-inflammatory/fibrotic phenotype at 20 weeks

**Figure 1:**
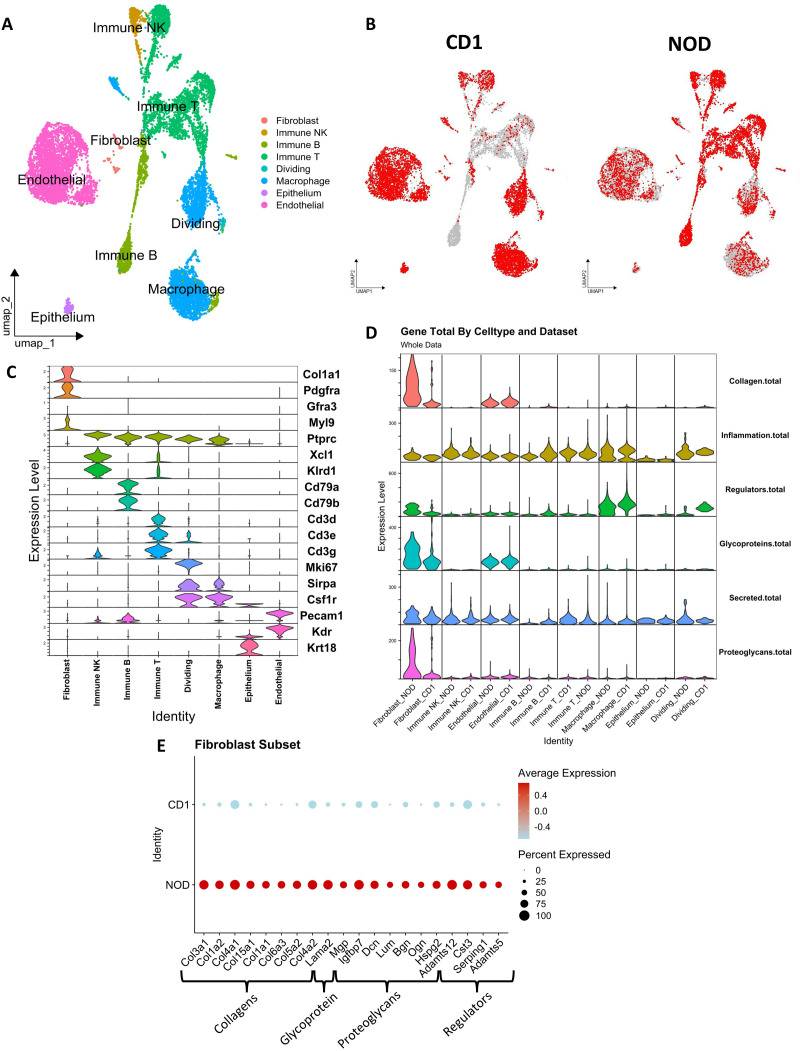
Single Cell RNA-seq analysis comparing CD1 mice and NOD/ShiLtJ mice at 20 weeks. **(A)** Uniform Manifold Approximation and Projection (UMAP) of the integrated dataset from CD1 and NOD/ShiLtJ female mouse SMG and SLG salivary glands (n = 4 mice of each strain). **(B)** A UMAP showing all cell contributions in red from the CD1 (left) or NOD (right) samples in the integrated dataset. **(C)** A violin plot showing key genes used to identify each cell cluster in the X axis. Fibroblasts are *Myl9*^+^, *Pdgfra^+^* and *Col1a1*^+^. The dividing cells are largely not fibroblasts but are primarily a mix of *Csf1r^+^*, *Sirpa*^+^, *Ptprc*^+^ macrophages and *CD3e*^+^ T cells. **(D)** A violin plot showing the expression of ECM and ECM-associated proteins in indicated clusters. The cell populations are separated by dataset of origin to allow for comparison of expression levels. **(E)** A dotplot illustrating the differences in the DEGs in the fibroblast cluster for each ECM category between CD1 and NOD/ShiLtJ salivary gland cells.

To determine if the NOD/ShiLtJ mouse develops a fibrotic response in the submandibular glands, female NOD/ShiLtJ mice were aged to 20 weeks, as previous studies have shown significant lymphocytic infiltration of the SMG, and onset of disease by 18 weeks (21). Glands were collected from naïve NOD/ShiLtJ and age-matched outbred, control mice (strain CD-1) at 20 weeks and subjected to cell dissociation and single-cell RNA sequencing (scRNAseq) to identify changes in key cell populations (**Figure 1A**). We observed changes in the amount of adaptive immune cells in the NOD/ShiLtJ as compared with the CD1 sample (**Figure 1B**), consistent with the increased lymphocytic infiltration previously reported. Established cell marker genes were used to identify and subcluster the cell populations for downstream analysis (21) (**Figure 1C**). When comparing the cell populations in NOD/ShiLtJ vs CD1 mouse salivary glands, we measured the abundance of transcripts for genes in inflammatory mediators and Matrisome categories (44) (**Figure 1D**). While many cell populations expressed genes encoding inflammatory mediators and secreted molecules, the fibroblasts from the NOD mouse salivary glands expressed significantly higher levels of specific collagens, Matrisome regulators, glycoproteins, and proteoglycans than fibroblasts from CD1 mouse salivary glands. Macrophages also expressed higher levels of ECM mRNAs in the NOD as compared to the CD1 SMG.

Focusing on mRNAs in the Matrisome list (44, 45) that were expressed by the fibroblasts, we found many specific transcripts encoding collagens, glycoproteins, proteoglycans, and Matrisome regulatory proteins that were present at higher levels in the NOD/ShiLtJ mouse SMGs relative to the CD1 SMGs, with a large percentage of the cells expressing most of the genes examined (**Figure 1E**). Additionally, we performed unbiased comparisons between fibroblasts and macrophages to identify potentially novel genes that are related to the progression of the NOD/ShiLtJ phenotype (**Supplementary Figures 1A, B**). Within the list of the top 15 differentially expressed genes (DEGs), many genes that have been reported to be increased in activated pro-fibrotic fibroblast populations are higher in the NOD fibroblasts than the CD1 fibroblasts, including *Fap, Col3a1*, and *Adamts12 (*46–48). *Mrc2*, which is known to facilitate collagen remodeling (49) and *Mndal* (50), an interferon (IFN)-stimulated gene, are also differentially expressed in the NOD mouse fibroblasts. *Mndal* was also differentially expressed by NOD macrophages as well as *Stat1*, which is expressed in macrophages tending towards an M1 phenotype following IFN stimulation. This analysis confirmed that fibroblast and macrophage gene expression differs in the NOD/ShiLtJ mouse strain relative to control mice and suggests that both cell types are in a myofibroblast like, pro-fibrotic/pro-inflammatory state in the NOD/ShiLtJ mouse.

### 3.2 The NOD/ShiLtJ salivary glands show a fibrotic response by 20 weeks

**Figure 2:**
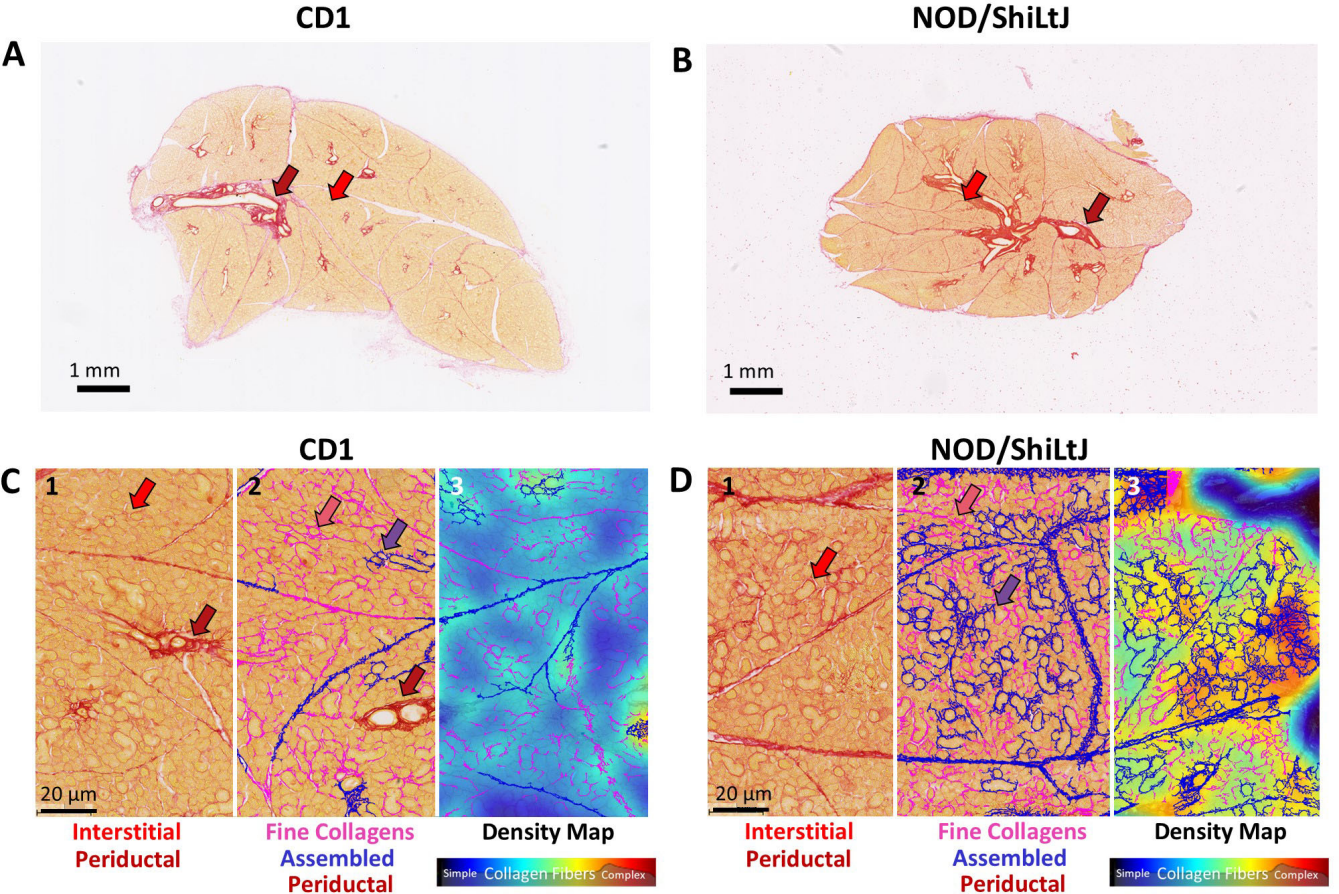
SMG of NOD/ShiLtJ mouse accumulates excess ECM. **(A)** tissue section of whole SMG from a 20-week CD1 female mouse reveals normal ECM accumulation around ducts and in interstitial regions when stained with PSR. Scale = 1 mm **(B)** A section of a whole SMG from a 20-week NOD/ShiLtJ mouse stained with PSR reveals excessive ECM accumulation in the interstitial periacinar (red, red arrow) and periductal (dark red) regions. Scale = 1 mm. **(C)** CD1 and **(D)** NOD/ShiLtJ SMGs stained with PSR demonstrating FibroNest quantification process on one slide with progressive overlays on each tissue panel (1-3). **(C1, D1)** PSR showing interstitial collagen around the acini (red arrow) and periductal collagen accumulating around ducts (dark red arrow). **(C2, D2)** FibroNest software detects fine collagens (pink arrow) and assembled collagens (indigo arrow) in architecture complexity color map. **(C3, D3)** PharmaFest architecture collagen complexity density map using look up table to indicate simple (blue) to complex (red) collagen. Scale, 20 µm.

To interrogate fibrosis, gland sections were subjected to PSR staining to detect fibrillar collagens. PSR staining showed increased fibrosis in the NOD/ShiLtJ SMG relative to control CD-1 (**Figures 2A, B C1, D1**). To more accurately quantify the periacinar fibrosis in the 20 week mice, we turned to PharmaNest’s FibroNest’s platform, which has been used to quantify multiple aspects of the fibrotic response in many contexts (43, 51–53). PharmaNest’s AI-driven quantification and interpretation methods (FibroNest) (43) were used to examine specific aspects of collagen deposition and arrangement from the PSR images by first detecting fine collagens and assembled collagens in the periacinar regions separately from the periductal regions (**Figures 2C2, D2**). Collagen density was also calculated and represented with a density map (**Figures 2C3, D3**). Quantification of 140 quantitative fibrotic traits (qFTs) from the fine collagens, assembled collagens and density maps (excluding the periductal collagen) revealed significantly increased trait scores in all 5 major categories (**Supplementary Figure 2**), revealing a fibrotic phenotype in the NOD/ShiLtJ SMG relative to control CD-1 glands. Most of the qFTs showed generally higher levels in the NOD/ShiLtJ SMG relative to control CD-1 glands (**Supplementary Figure 2**). These scores generally reveal increases in fine collagen, thick collagen, and complex collagen architecture, suggestive of higher collagen levels and increased crosslinking, revealing interstitial fibrosis in the NOD/ShiLtJ SMGs at 20 weeks.

### 3.3 The fibrotic response in NOD/ShiLtJ salivary glands correlates with hyperglycemia

**Figure 3:**
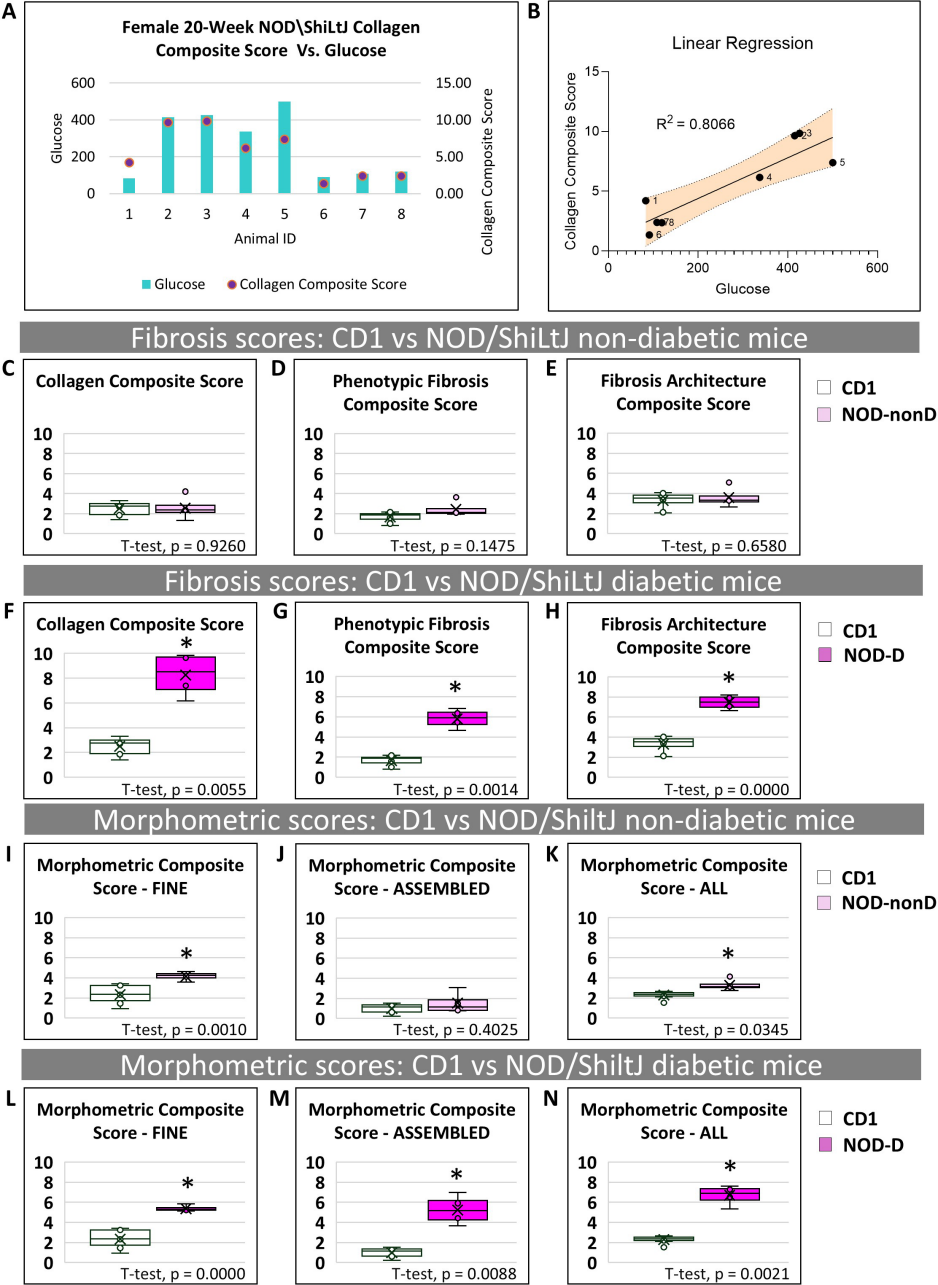
The diabetic phenotype in the NOD/ShiLtJ mouse correlates with the fibrotic phenotype. **(A)** FibroNest Collagen Composite Score compared to glucose reading from 20-week-old female NOD/ShiLtJ SMG. **(B)** Linear regression analysis demonstrates the relationship between glucose and Collagen Composite Score with R^2^ = 0.8066. Shaded area shows the 95% confidence intervals. **(C–N)** Selected qFT scores from **Supplementary Figure 2** were compared separately in SMGs from 20 week female mice: CD1 vs female diabetic NOD/ShiLtJ (NOD-D) (glucose > 250, n = 4) and Non-Diabetic (NOD-Non-D) Phenotype (glucose < 250) **(C–E)** qFT fibrosis Scores for CD-1 vs NOD-nonD. **(C)** Collagen Composite Score, **(D)** Phenotypic Fibrosis Composite Score, **(E)** Fibrosis Architecture Composite Score. **(F–H)** qFT Fibrosis Scores for CD-1 vs NOD-D **(F)** Collagen Composite Score, **(G)** Phenotypic Fibrosis Composite Score, **(H)** Fibrosis Architecture Composite Score. **(I–K)** qFT Morphometric Composite Scores for CD-1 vs NOD-nonD SMGs. **(I)** Morphometric Composite Score – FINE, **(J)** Morphometric Composite Score – ASSEMBLED, and **(K)** Morphometric Composite Score – ALL. **(L–N)** qFT Morphometric Composite Scores for CD-1 vs NOD-D SMGs. **(L)** Morphometric Composite Score – FINE, **(M)** Morphometric Composite Score – ASSEMBLED, and **(N)** Morphometric Composite Score – ALL. Y axis in all graphs represents arbitrary units. CD-1 n=8 mice. NOD/ShiLtJ n= 8 mice. n = 4 mice each subgroup (Diabetic and non-diabetic); statistical analysis performed by Student’s t-test. p values are indicated. *p < 0.05.

When examining fibrosis phenotypes (**Supplementary Figure 2**), we noted there seemed to be two phenotypes in the NOD/ShiLtJ mice. Whereas all 8 of the CD-1 mice showed an overall normal phenotype (**Supplementary Figure 2**), four of the NOD mice showed a fibrotic phenotype (NOD2-5), three showed a normal phenotype (NOD6-8) and one an intermediate phenotype (NOD1). Since NOD/ShiLtJ mice are diabetic (24), we assessed the diabetic phenotype by taking blood glucose measurements. Animals with glucose readings >250 mg/dL (54, 55) were labeled as phenotypically diabetic (NOD-D) and those that were lower were labeled as not diabetic (NOD-nonD). To determine if the fibrotic phenotype is related to the diabetic phenotype, we compared the glucose score of the NOD/ShiLtJ animals with the collagen composite score qFT. We observed a correlation between the collagen composite score and the glucose level, which we quantified with linear regression (**Figures 3A, B**). Given this, we reanalyzed the data in **Supplementary Figure 2**, separating the non-diabetic (NOD-nonD) mice from the diabetic (NOD-D) mice for comparisons of the FibroNest scores. The NOD-nonD phenotype did not show a significant difference in most of the FibroNest fibrotic scores when compared to the control CD1 (**Figures 3C–E, I–K**). In contrast, the diabetic NOD-D SMGs showed a statistical increase in all FibroNest scores when compared to control CD1 (**Figures 3F–H, L–N**). These data indicate that the fibrosis in the NOD/ShiLtJ mice is correlated with the diabetic phenotype.

### 3.4 Nintedanib modulates collagen assembly in the NOD/ShiLtJ mouse submandibular glands

**Figure 4:**
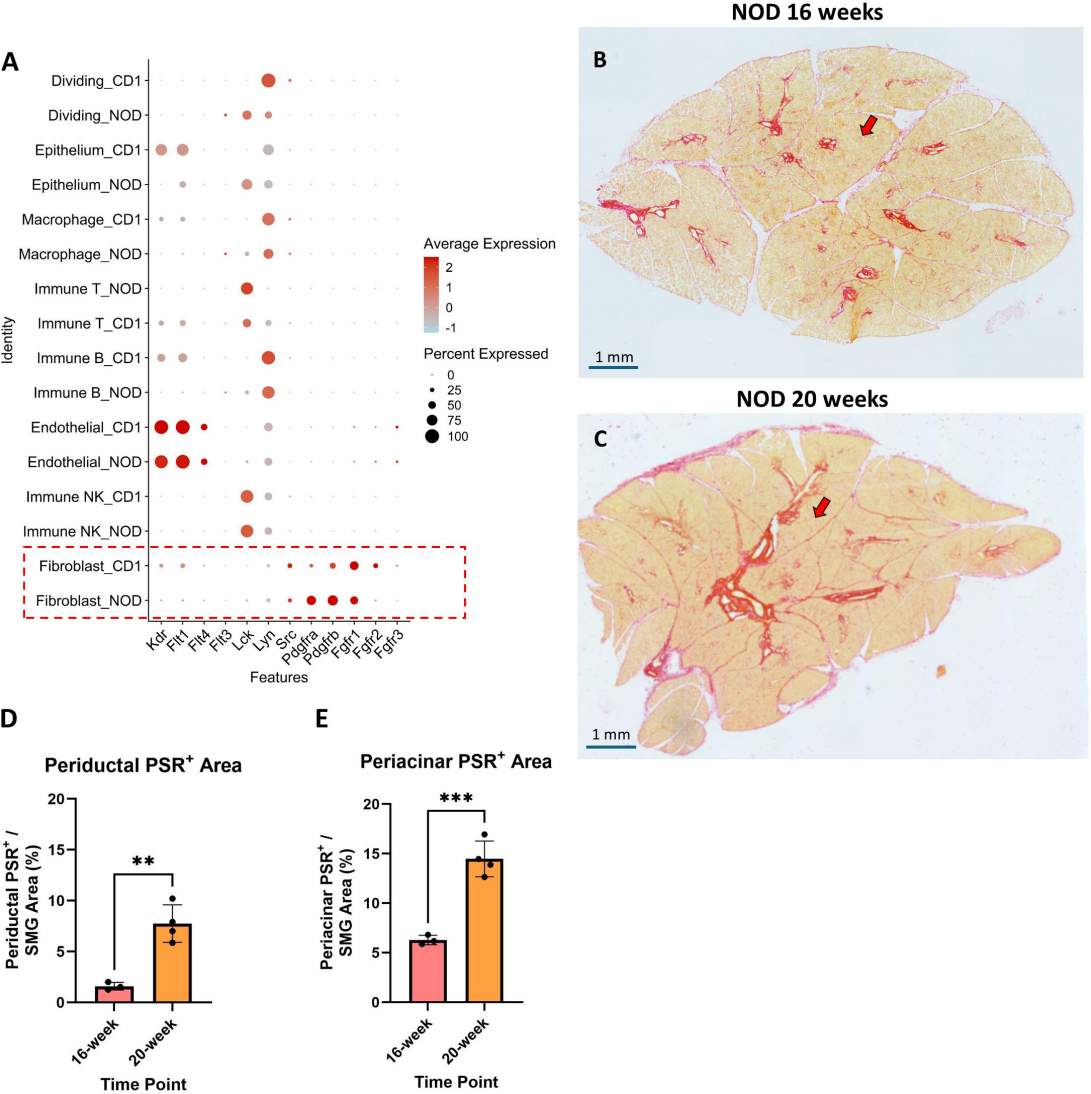
Expression of nintedanib target genes in fibroblasts of NOD/ShiLtJ and CD1 SMG from scRNASeq. **(A)** Dot plot showing the expression levels of known nintedanib targets in 20-week-old female SMGs from CD1 and NOD/ShiLtJ mice by cell clusters with fibroblasts indicated (red dotted line). **(B)** 16-week-old NOD/ShiLtJ SMG and SLG stained with PSR. **(C)** 20-week-old NOD/ShiLtJ SMG and SLG stained with PSR. Red arrow shows ECM accumulation in an interstitial periacinar region. Scale, 1 mm. **(D)** Quantification of periductal PSR stain as percentage of total tissue area. **(E)** Quantification of periacinar PSR stain as percentage of total tissue area. 16-week n=3 mice, 20-week n=4 mice. Unpaired t-test **p < 0.01, ***p < 0.001.

**Figure 5:**
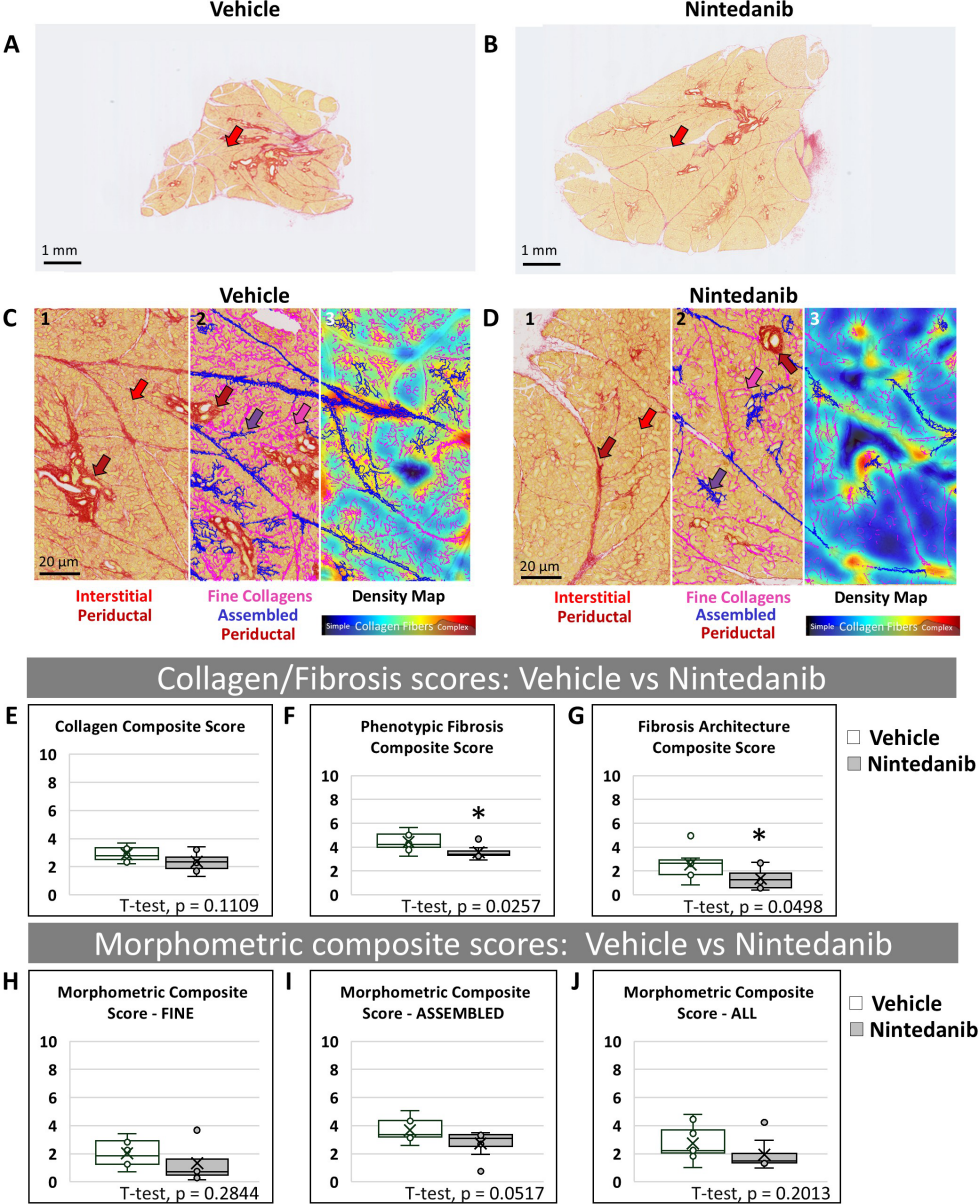
Nintedanib treatment in NOD/ShiLtJ Mice shows partial reduction in fibrotic characteristics. SMG stained with PSR from a 24-week-old female NOD/ShiLtJ mice treated with **(A)** Vehicle or **(B)** Nintedanib. Scale, 1 mm. Red arrow shows accumulation of ECM in interstitial periacinar regions. **(C)** Vehicle and **(D)** Nintedanib-treated NOD/ShiLtJ SMGs at 24 weeks stained with PSR demonstrating FibroNest quantification process on one slide with progressive overlays on each tissue panel (1-3). Scale, 20 µm. **(C1, D1)** PSR stain showing interstitial collagen (red arrow) and periductal collagen (dark red arrow) **(C2, D2)** detection of fine collagen (pink arrow), assembled collagen (indigo arrow) with periductal collagen (dark red arrow), which was excluded from quantification. **(C3, D3)** Pharmanest architecture collagen complexity density map using look up table to indicate simple (blue) to complex (red) collagen. **(E–J)** Selected qFT fibrosis metrics from **Supplementary Figure 3** for vehicle vs nintedanib**-**treated SMGs. **(E)** Collagen Composite Score, **(F)** Phenotypic Fibrosis Composite Score, **(G)** Fibrosis Architecture Composite Score, **(H–J)** Selected qFT morphometric composite scores: **(H)** Morphometric Composite Score – FINE, **(I)** Morphometric Composite Score – ASSEMBLED, and **(J)** Morphometric Composite Score – ALL. Y axis in all graphs for each treatment. Statistical analysis performed by Student’s *t*-test. *p* values are indicated. * p < 0.05.

We then tested the antifibrotic small molecule, nintedanib, for reduction of fibrosis in the SMGs of the NOD/ShiLtJ mice. We first confirmed using scRNASeq data that the nintedanib targets are expressed in the glands (**Figure 4A**). We noted increased expression of*, Pdgfra and Pdgfrb* in the NOD/ShiLtJ fibroblasts relative to control CD1. We evaluated the progression of fibrosis in the NOD/ShiLtJ mice to identify an early fibrosis stage to administer nintedanib. Fibrosis is detectable but very low in the salivary glands of the female NOD mouse at 12 weeks (pre-disease) and increases over time (**Supplementary Figure 3**) (25). Comparison of the NOD/ShiLtJ SMGs stained with PSR at 16 weeks (mid stage disease) and 20 weeks (late stage disease) showed increased fibrosis at 20 weeks (**Figures 4B, C**), and quantification based on thresholding to generally select fibrotic responses in these two regions of tissue (**Supplementary Figure 4**) revealed that both periductal fibrosis and periacinar fibrosis are present at lower levels in 16 week SMG relative to 20 week SMG (**Figures 4D, E**). We thus selected 16 weeks as the timepoint to start nintedanib treatments to mimic patients at an early/mid stage of disease. In previous work, doses of 30–100 mg/kg of body weight were shown to be effective in mice with IPF (30), systemic sclerosis (32), and liver fibrosis (33). We thus began treatment with nintedanib at 16 weeks in 10 female mice for each cohort using a dose of 1.5 mg/mouse (68 mg/kg) of nintedanib or vehicle every other day via oral gavage until 24 weeks when SMGs were harvested and stained with PSR (**Figures 5A, B**).

Images of SMG PSR-stained cryosections were then quantified with FibroNest scoring to evaluate the periacinar fibrotic response following nintedanib treatment. PharmaNest’s AI quantification and interpretation methods (FibroNest) (43) were used to examine specific aspects of collagen deposition and arrangement from the PSR images by first detecting fine collagens and assembled collagens after treatment with vehicle or nintedanib (**Figures 5C, D**), and Quantitative fibrotic traits (qFTs) were evaluated (**Supplementary Figure 5**). Examination of the qFTs previously identified in the NOD/ShiLtJ mouse SMGs (**Figures 3F–H, L–N**), revealed statistical differences in Phenotypic Fibrosis Composite Score (**Figure 5F**) and Fibrosis Architecture Composite Score (**Figure 5G**) in nintedanib-treated glands relative to Vehicle-treated glands. The collagen composite score and morphometric scores for nintedanib-treated SMGs vs vehicle-treated glands were not statistically different (**Figures 5E, H–J**). However, the nintedanib-treated animals showed lower numbers for several qFTs in **Supplementary Figure 5**, including collagen branch counts, collagen areas and perimeters, entropy, and eccentricity counts for assembled collagen. These qFTs reveal modest but measurable decreases in fibrosis progression in female SMGs following 8 weeks with nintedanib treatment relative to vehicle-treated controls.

## 4 Discussion

Fibrosis is a component of many diseases, and the changes in the ECM that occur with fibrosis can significantly alter homeostasis by disrupting signaling, leading to increased cellular stress, inflammation, and organ dysfunction. Using quantitative image-based analysis, we show that female NOD/ShiLtJ salivary glands have both a periductal and a periacinar fibrotic phenotype that develops over 20 weeks with disease progression. Further, we show that the fibrotic response is heterogeneous, which mirrors the heterogenous nature of the fibrotic response in patients (20). Of note, the degree of assembled collagen in the periacinar region of the SMGs in female NOD/ShiLtJ mice correlates with the diabetic phenotype, as previously indicated (25). Treatment with the FDA-approved fibrotic inhibitor, nintedanib, caused a modest reduction in periacinar fibrosis in the affected salivary glands. While the nintedanib-treated animals showed persistent collagen by most criteria, the phenotypic fibrosis composite score, fibrosis architecture score, and morphometrics composite score – assembled, were all lower in in the periacinar regions of nintedanib-treated animals. While nintedanib did not eliminate or prevent fibrosis, these qFTs suggest that nintedanib disrupted fibrotic network organization and reduced the advanced collagen maturation in the periacinar region that occurs with progressive fibrosis. These findings are consistent with nintedanib interfering with more complex and crosslinked collagen assembly, consistent with electron microscopy studies (56) showing decreased collagen assembly in primary human fibroblasts from idiopathic pulmonary fibrosis patients.

Chronic hyperglycemia stimulates signaling pathways that drive fibrosis across many organs. Downstream changes in the renin-angiotensin-aldosterone system and advanced glycation end products (AGEs) have been reported as well as increased concentrations of growth factors including TGF-β (57). Patients with type I diabetes often present with fibrosis in the pancreas and other organs (58). Our data suggests that in the NOD/ShiLtJ salivary glands, hyperglycemia is likely a primary driver of fibrosis. However,. Hwang et al, proposed that the Th17/IL-17 axis is the primary driver of inflammation and fibrosis in the NOD mouse, with diabetes acting as an exacerbating metabolic driver (25). Importantly, the heterogeneity in nintedanib response observed in our study may reflect the variable age of onset of hyperglycemia across individual animals, further supporting the contribution of glycemic state to fibrotic severity.

Our scRNASeq data analysis of DEGs reveals a transcriptional profile consistent with activated, pro-fibrotic fibroblasts, or myofibroblasts, in the NOD mouse. *Fap* (fibroblast activation protein) is a canonical marker of activated fibroblasts in salivary glands and other organs and is known to promote macrophage activation, ECM turnover and fibroblast proliferation. *Adamts12*, recently identified as a fibroblast-specific gene that is increased in kidney and heart fibrogenesis, can remodel the ECM to facilitate activation and migration of injury-responsive fibroblasts. The high expression of *Mndal* in fibroblasts and macrophages, an interferon-inducible gene that is implicated in autoimmune susceptibility including lupus (50), is consistent with an interferon signature and participation of the fibroblasts in immune dysregulation. The fibroblasts also show increased expression of multiple collagens (e.g. *Col1a1, Col1a2, Col3a1*, and *Col5a2*), consistent with broad increases in ECM level in the glands. Overall, these data suggest that fibroblasts adopt both a pro-inflammatory and pro-fibrotic phenotype, which may be restricted to different cell sub-populations, similar to observations in human patients (59). Our scRNASeq data also reveals expected increases in B and T cell populations, similar to prior reports for the similar NOD.B10 mouse strain that lacks the diabetes co-morbidity (60). Previous reports in the NOD.B10 mouse strain indicate that the epithelial cells upregulate interferon signaling genes, including *Irf7, Cxcl10, Bst-2*, and *Stat1*, suggesting that epithelial cells also contribute to interferon signaling during SjD disease progression in the NOD.B10 mouse.

The ability to assess the correlation of the fibrotic response with gland function has been controversial and hindered by a lack of quantitative tools (61). Although it would be highly valuable to evaluate the contribution of anti-fibrotic inhibitors to functional differences in saliva output, we were unable to measure meaningful nintedanib-dependent increases in saliva levels due to high heterogeneity in our measurements. Other studies have nevertheless correlated fibrotic state with saliva levels. In one study, quantification of H&E-stained tissues associated minor salivary gland fibrosis with focus score, independent of aging (20). In recent work, Relative Interstitial Fibrosis Area (RIFA) was quantified from trichrome-stained minor salivary glands from SjD patients (62). With this analysis, a correlation was demonstrated between unstimulated saliva flow and RIFA in minor salivary glands. As with the RIFA method, in this study we quantified the interstitial area near the acini where the ECM is most likely to affect gland secretory function. Here we demonstrate the utility of the more sensitive FibroNest analysis of PSR staining (63, 64), which has a higher signal to noise ratio than trichrome staining, enabling a more detailed analysis of the collagen assembly state in the periacinar region. Although periductal fibrosis is excluded from FibroNest quantification, future quantification of PSR staining of the periductal staining pattern though AI-enhanced image analysis will likely be informative given the increases in periductal fibrosis from 16–20 weeks in the NOD/ShiLtJ mouse SMG.

The digital pathology approach and FibroNest Scores used here are not without their limitations. The composite scores may be subject to overfitting, given the limited sample sizes and absence of independent validation cohorts. Spatial heterogeneity of tissue may also introduce sampling variability that limits the utility of phenotypic scores. Nevertheless, recent data in liver cirrhosis indicate that the sampling variability of phenotypic scores is relatively low, with an average coefficient of variation of 16% (43). Given this, the robustness and consistency of the PSR protocol and high signal to noise relative to trichrome staining, FibroNest quantification of fibrosis may have utility for future SjD studies using animal models and human patient samples, similar to other conditions (43, 51, 52, 65–67).

SjD and type 2 diabetes share overlapping inflammatory and immune dysregulation pathways. However, diabetes is not among the common comorbidities in SjD patients, and data on the prevalence of diabetes in SjD patients remains limited. Regardless, once the relationship between diabetes and fibrosis is better understood, our data suggest that knowledge of patient glycemic state may be beneficial in guiding use of anti-fibrotic drugs for treatment of SjD. These data also point to the possibility that different pathways drive distinct fibrotic states, and that more comprehensive knowledge of the mechanisms underlying fibrotic phenotypes will be essential for the rational use of anti-fibrotic agents in patients. Fibrotic inhibitors have been approved by the FDA for several diseases and have shown limited efficacy in reducing the fibrotic burden and improving organ function. Nintedanib has demonstrated efficacy in several lung diseases, including patients with ILD (31), IPF (68) and COVID19 (69) but has only been tested in salivary glands of human patients for salivary gland cancer (70). Although ILD occurs in roughly 10–20% of primary Sjögren’s patients by clinical detection (71), SjD patients were not separately analyzed in the INBUILD ILD nintedanib trial. The effects of nintedanib are complex because it is a broad-spectrum tyrosine kinase inhibitor affecting multiple targets, not all of which are necessarily involved in fibrosis. Fibroblasts are altered in SjD, transitioning to immunofibroblasts (72) and modulating extracellular matrix organization (59). Other growth factors beyond nintedanib’s primary targets have been implicated in fibrosis, including Transforming Growth Factor beta (TGFβ), which is a major driver of fibrosis in many organs (73) and in salivary gland fibrosis models (36, 74), although nintedanib may decrease TGFβ-mediated fibrosis indirectly. Comparison of the genes increased in fibroblasts of patients having IPF and SjD identified only *Setd8* and *Cxcl12* as common fibroblast genes in both diseases (62), suggesting that the fibrotic responses in IPF and SjD are likely distinct, indicating a requirement for personalized therapeutic approaches across patients. Transcriptomic and proteomic approaches to identify differentially expressed genes and proteins may provide insights into additional targets for the treatment of fibrosis in SjD and other fibrotic diseases. Analysis of the fibrotic response and its relationship to inflammation will require validation with larger sample sizes and evaluation in human patients.

In summary, the findings of this study highlight the utility of the NOD/ShiLtJ mouse in the study of fibrotic responses and provide evidence that fibroblast-driven fibrotic remodeling contributes to glandular pathology in a SjD-like disease. While nintedanib yielded only a limited benefit in this study, the modest reduction in periacinar collagen maturation points to fibrotic network organization as a tractable therapeutic target. Emerging strategies that limit the fibroblast-immune crosstalk offer a promising avenue for future investigation. Nerandomilast, the latest anti-fibrotic drug to be approved by the FDA (75, 76), targets phosphodiesterase-4B, to suppress downstream fibrotic signaling. This drug works through targeting cyclic AMP-dependent pathways to reduce TGFβ-induced myofibroblast activation, collagen gene transcription, ECM accumulation, and cytokine release, opening additional non-receptor-targeted pathways for possible future therapeutics. The use of advanced AI-assisted digital pathology approaches for fibrosis assessment will aid in the future evaluation of anti-fibrotic mediators as therapeutics in SjD and other fibrotic diseases.

## Data Availability

The datasets presented in this study can be found in online repositories. The names of the repository/repositories and accession number(s) can be found below: https://www.ncbi.nlm.nih.gov/geo/, GSE314772.
