# Supplementary material for: Fibrotic remodeling in the NOD/ShiLtJ mouse model of Sjögren’s disease: insights from single-cell transcriptomics and AI-driven ECM quantification
Source: Front Immunol. 2026 Jul 20;17:1779014. doi: 10.3389/fimmu.2026.1779014 (PMC13429612; doi:10.3389/fimmu.2026.1779014)
Supplement: Supplementary file 1 [file DataSheet1.pdf]

Supplementary Figures Morrissey et al. 2026

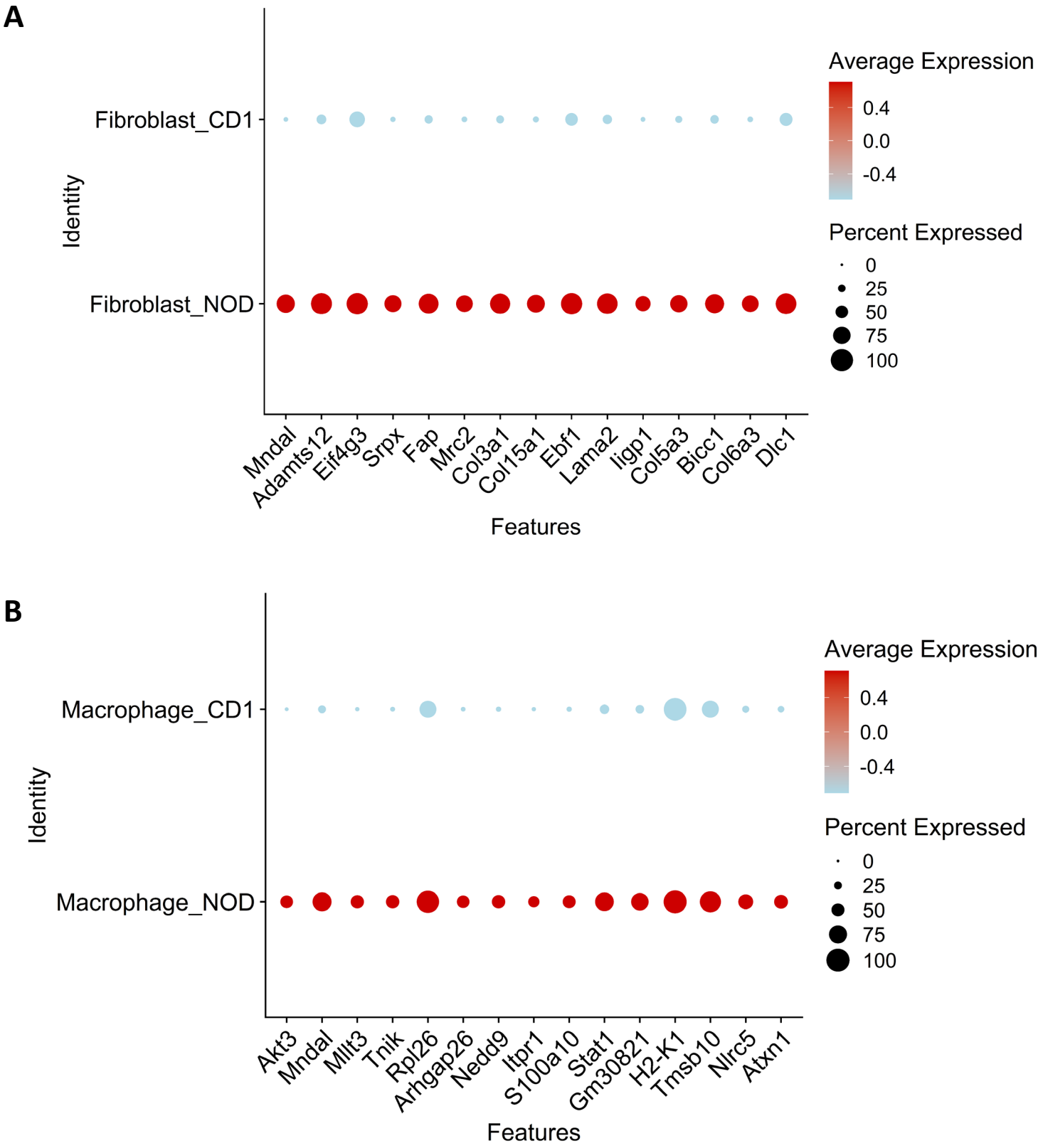

Supplemental Figure 1. Differentially expressed genes in fibroblasts and macrophages of the mouse submandibular salivary gland in the NOD vs CD1 mouse. **A.** Dot plot shows Top 15 DEGs in NOD vs CD1 Fibroblasts and **B.** Dotplot shows Top 15 DEGs in NOD vs CD1 macrophages. The lookup table shows the degree of expression while the dot size shows the percentage of cells that express the gene. Many genes that are known to be increased in activated pro-fibrotic fibroblast populations are higher in the NOD fibroblasts than the CD1 fibroblasts, including *Fap*, *Col3a1*, and *Adamts12*. *Mrc2*, which is known to drive collagen remodeling and *Mndal*, an interferon (IFN)-stimulated gene, are also differentially expressed in the NOD mouse. In the macrophages, Stat1, which is activated by IFN and TLR signaling to promote an M1 macrophage phenotype, is highly increased. *Mndal* is also highly expressed in the macrophage population.

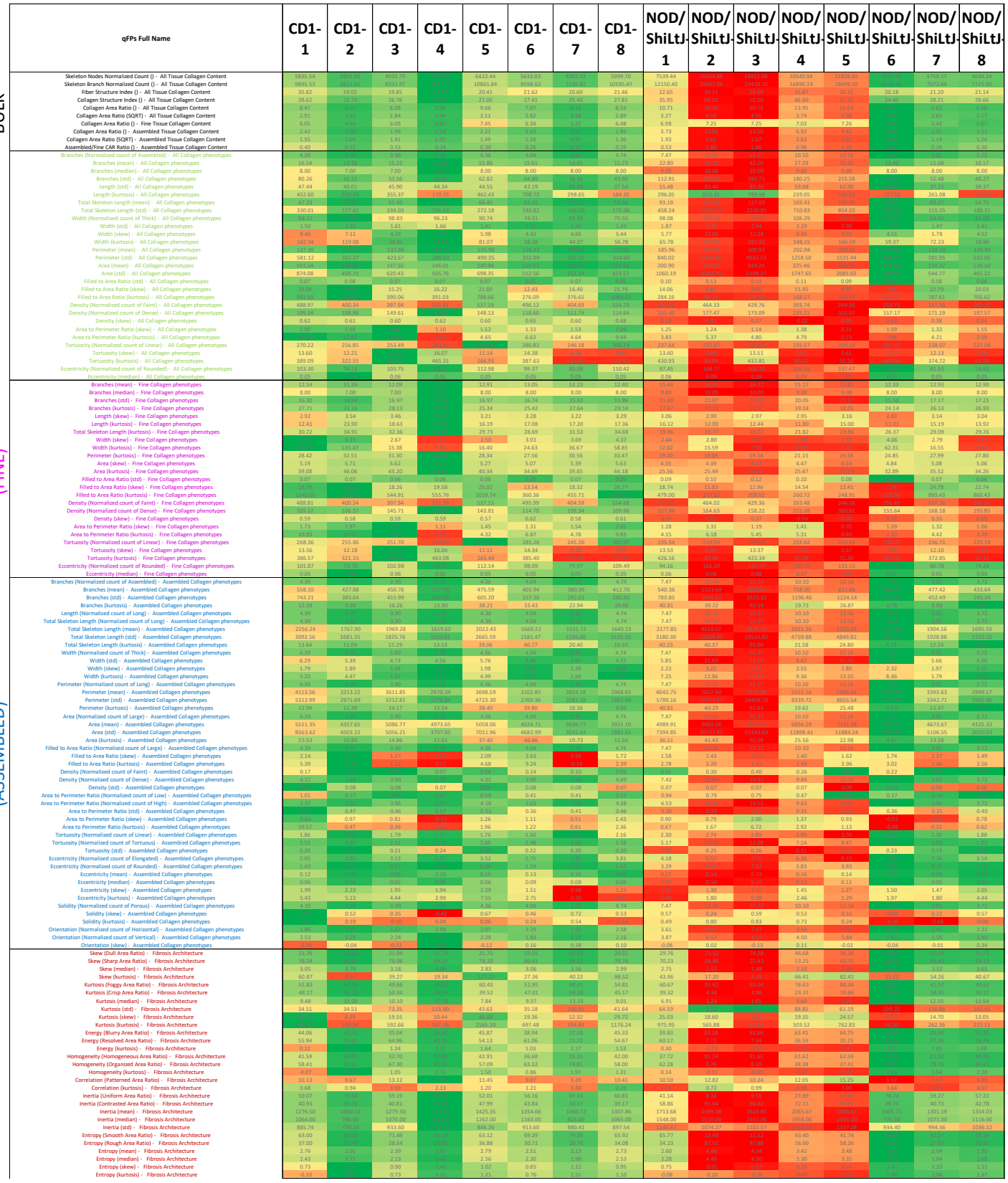

Supplementary Figure 2. FibroNest™ Heat Map for qLTs: CD1 compared to NOD/ShiLtJ SMGs. All values for quantitative traits are shown (Y axis) with each animal shown in a column with relatively low levels shown in green, high levels in red and zero in yellow. The color scheme is normalized within each qFT (row), so colors represent relative differences rather than absolute values. CD1 n=8, NOD/ShiLtJ n=8

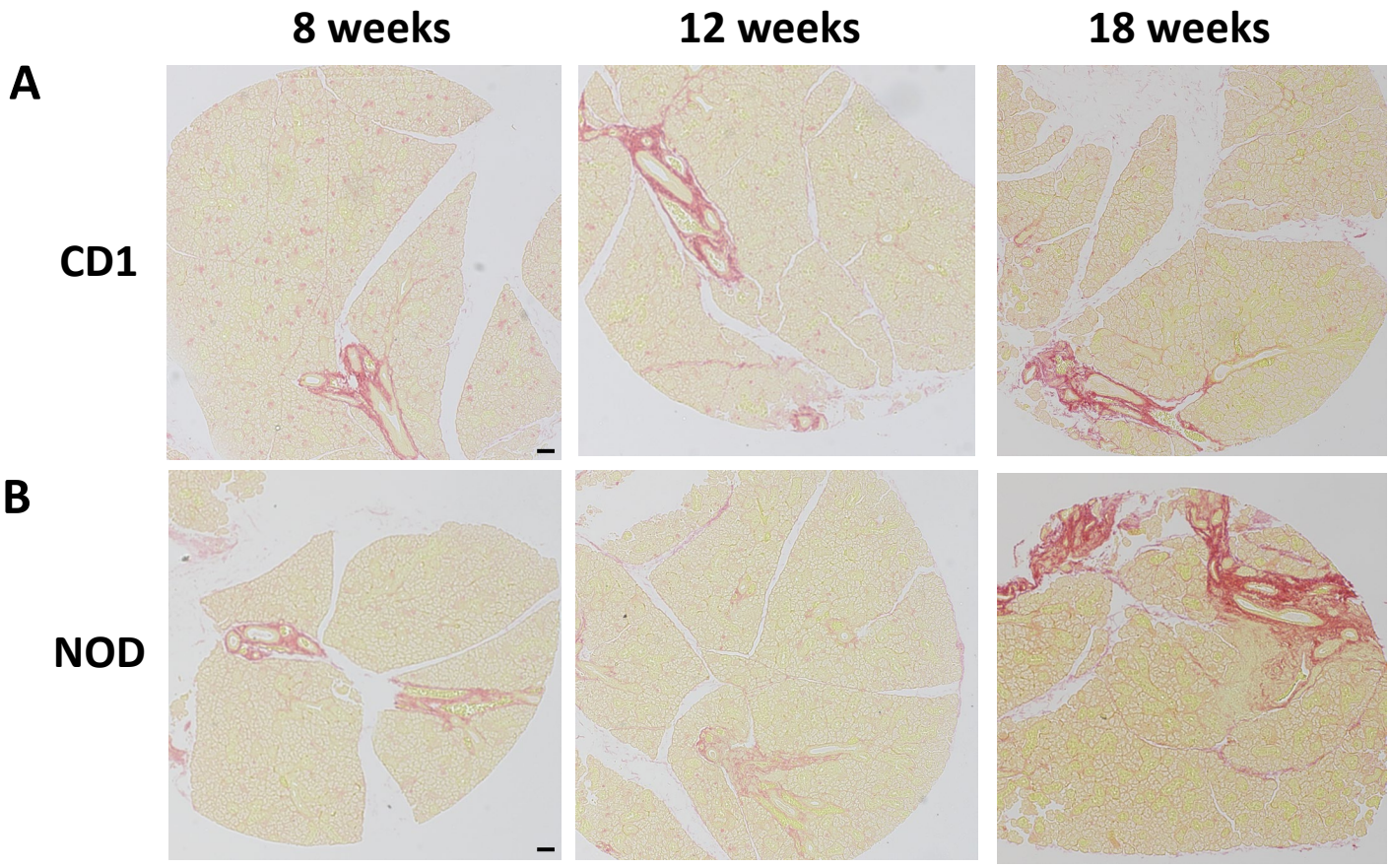

Supplementary Figure 3. Development of fibrosis over time in the female NOD mouse. A tissue microarray containing biopsy punches from **A.** CD1 and **B.** NOD female mouse SMGs at 8, 12, and 18 weeks were stained for PSR. Representative images are shown. Scale bars, 20 µm.

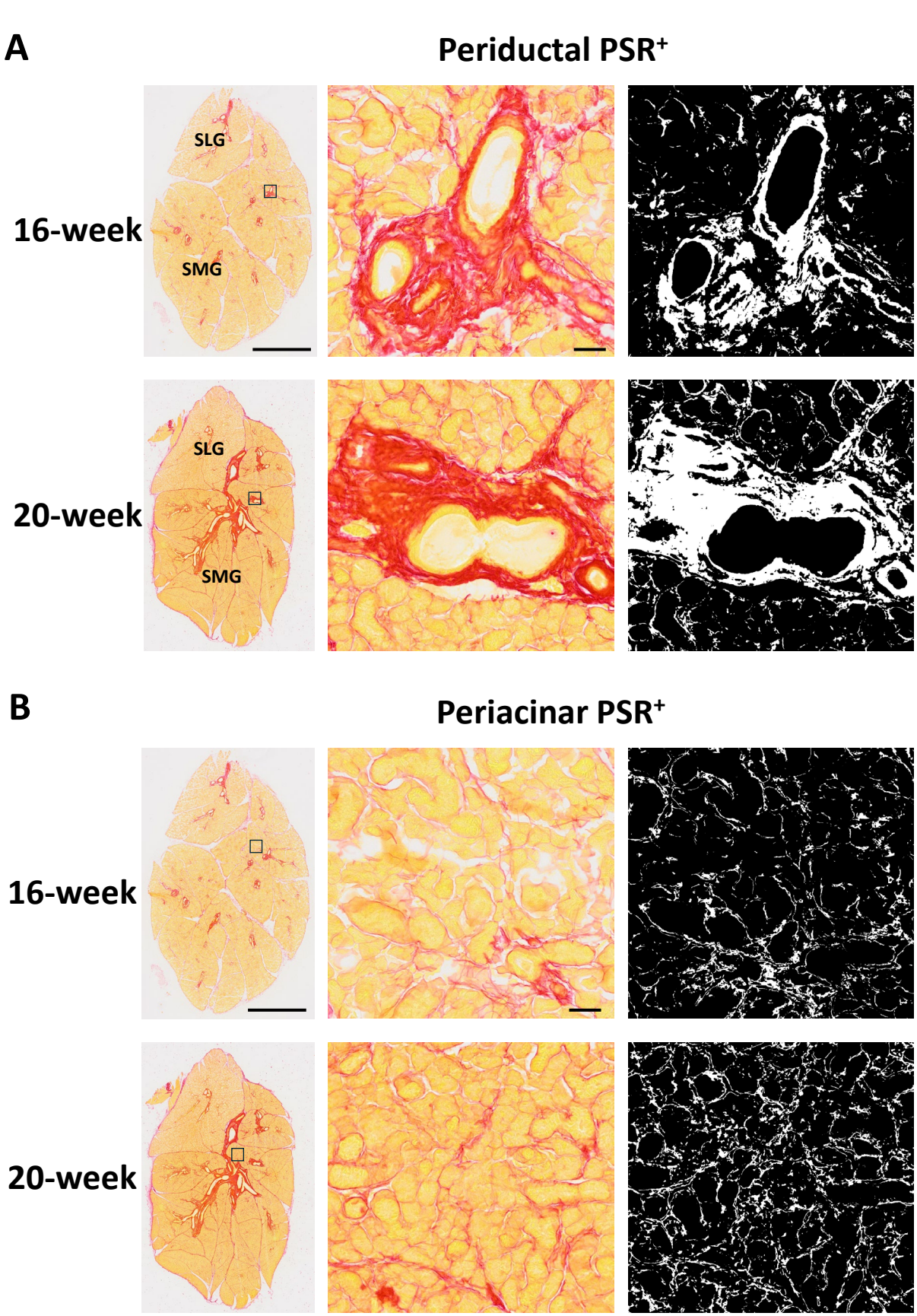

Supplementary Figure 4. Periductal and periacinar fibrosis increase in the female NOD mouse SMG from 16 to 20 weeks. Cryosections from female NOD mice at 16 (N=3) and 20 (N=4) weeks were stained with PSR and then quantified using FIJI. Thresholds were set to capture: **A** periductal fibrosis or **B** periacinar fibrosis in 16-week and 20-week female NOD mice. Representative whole glands are shown in the first column. Cropped PSR images of the region included in the black outline are shown in the second column.. Example masks showing the thresholded region that was quantified in Figure 4 are shown in the third column. Scale bar, 2 mm, first column and 10 µm, second column).

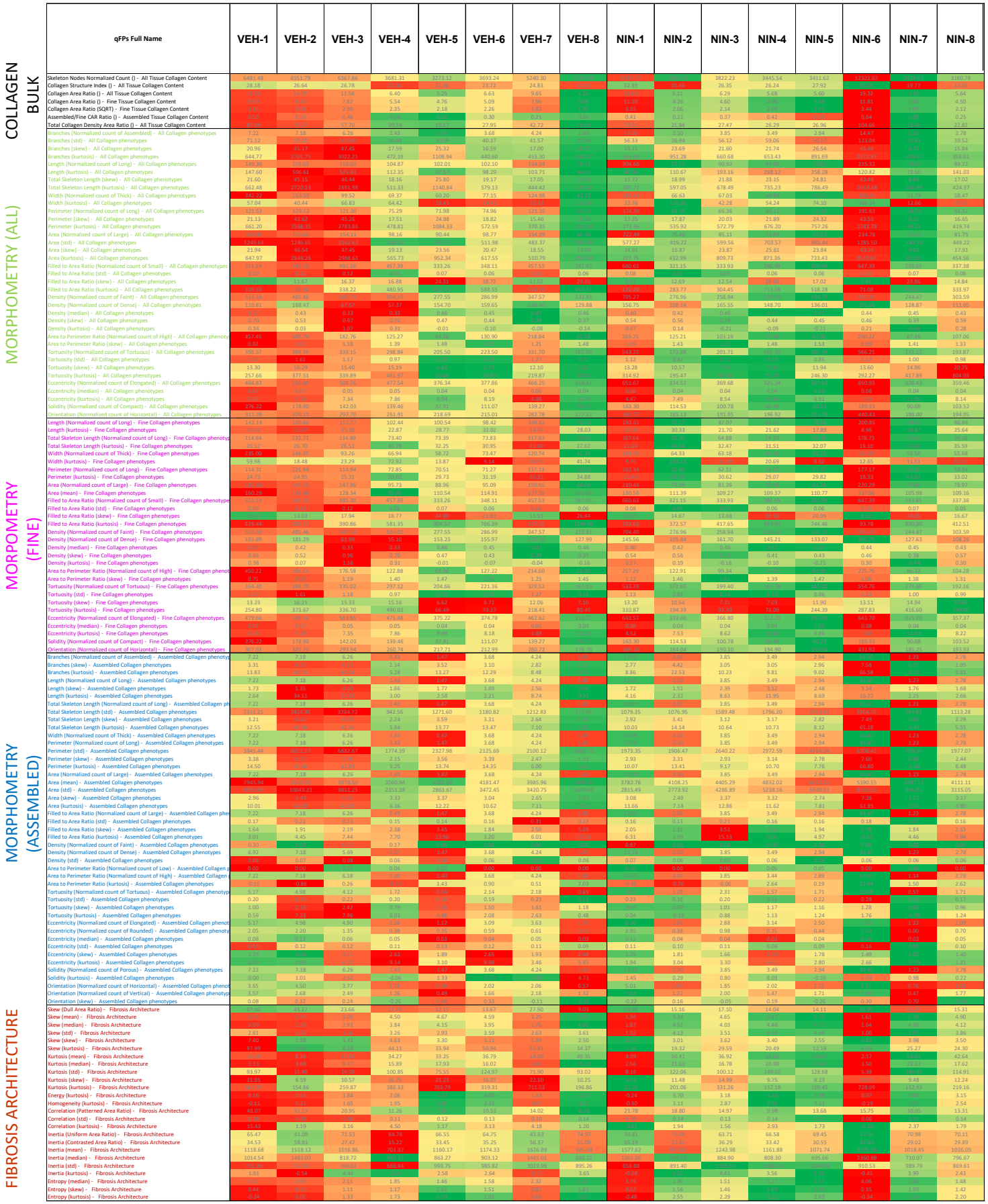

Supplemental Figure 5. FibroNest™ Heat Map for qLTs: NOD/ShiLtJ 24 Week Vehicle-treated compared to NOD/ShiLtJ Nintedanib-treatment SMGs. All values for quantitative traits are shown (Y axis) with each animal shown in a column with low levels shown in green, high levels in red and zero in yellow. The color scheme is normalized within each qFT (row), so colors represent relative differences rather than absolute values. Vehicle-treated n=8, Nintedanib-treated n=8
